# Evaluation of the *in vitro* and *in vivo* inhibitory effects of *Artemisia herba-alba* against the growth of piroplasm parasites

**DOI:** 10.5455/javar.2022.i592

**Published:** 2022-06-26

**Authors:** Rasha Eltaysh, Mohamed Abdo Rizk, Shimaa Abd El-Salam El-Sayed, Khaled Abouelnasr, Abdelnaser Ahmed Abdallah, Ikuo Igarashi

**Affiliations:** 1 National Research Center for Protozoan Diseases, Obihiro University of Agriculture and Veterinary Medicine, Obihiro, Japan; 2 Department of Pharmacology, Faculty of Veterinary Medicine, Mansoura University, Mansoura, Egypt; 3 Department of Internal Medicine and Infectious Diseases, Faculty of Veterinary Medicine, Mansoura University, Mansoura, Egypt; 4 Department of Biochemistry and Chemistry of Nutrition, Faculty of Veterinary Medicine, Mansoura University, Mansoura, Egypt; 5 Department of Surgery, Anesthesiology and Radiology, Faculty of Veterinary Medicine, Mansoura University, Mansoura, Egypt; 6 Veterinary Teaching Hospital, Faculty of Veterinary Medicine, Mansoura University, Mansoura, Egypt

**Keywords:** *Babesia*, *Theileria*, *Artemisia herbaalba*, *in vitro*, *in vivo*, combination therapy

## Abstract

**Objective:**

The effect of *Artemisia herba-alba* methanolic extract monotherapy and combination therapies on the *in vitro* growth of several *Babesia *and Theileria parasites *in vitro *and mice was investigated in this study.

**Materials and Methods:**

Fluorescence assay using SYBR Green I stain was used to evaluate the antibabesial efficacy inhibitory of *A. herba-alba* either *in vitro* or *in vivo.* Hematological parameters in the treated mice were analyzed using a Celltac MEK-6450 computerized hematology analyzer.

**Results:**

*Artemisia herba-alba* reduced the growth of *Babesia bovis*, *Babesia bigemina*, *Babesia divergens*, *Theileria equi*, and *Babesia caballi in vitro* in a dose-dependent manner. The *in vitro* inhibitory impact of *A. herba-alba* on *B. divergens* and *B. caballi* cultures was amplified when combined with either diminazene aceturate (DA). In *B. microti*-infected mice, a combination therapy consisting of *A. herba-alba* and a low DA dose inhibited *B. microti* growth significantly (*p* < 0.05) better than treatment with 25 mg kg^−1^ DA.

**Conclusions:**

These data show that *A. herba-alba*, when paired with a modest DA dose, could be a promising medicinal plant for babesiosis treatment.

## Introduction

*Babesia* and *Theileria* are tick-borne parasites that infect animals’ erythrocytes, causing enormous economic losses in the agricultural industry and worldwide trade [1,2]. Clinical indicators of this infection include fever, malaise, jaundice, hemoglobinuria, and death [3,4]. The infection is mainly caused by either *Babesia bovis* (*B. bovis*) and *Babesia bigemina* (*B. bigemina*) in cattle [3] or *Theileria equi* (*T. equi*) and *B. caballi* in horses [5]. Because the inhibitory effects of recently developed antibabesial drugs should be evaluated in laboratory animals before they are used in the field, and because there are no acceptable laboratory experimental animals for bovine and equine Babesia infections, a rodent Babesia model infected with *B. microti* or a gerbil infected with *Babesia* *divergens* is used for drug evaluation [6,7].

For many years, the standard therapies for babesiosis were diminazene aceturate (DA) and imidocarb dipropionate [8,9]. However, they have significant drawbacks, such as a long time to remove tissue, toxicity, and, in the case of DA, unavailability in certain regions [9]. Furthermore, new research has revealed that Babesia parasites may develop DA resistance [10,11]. As a result, finding more effective and safer antipiroplasm drugs has become a top objective. Natural phytochemicals could be a potential alternative in this scenario. In the same vein, *Artemisia herba-alba*, commonly known as desert or white wormwood, is used in folk medicine to treat various diseases [12,13]. Several studies have reported the wide pharmacological activities of *A. herba alba* as antidiabetic, antimicrobial, antimalarial, acaricidal [12,14,15], anticancer, and antioxidant [16,17]. However, *A. herba-alba* extract’s antibabesial efficacy is yet to be determined. As a result, in the current investigation, we evaluated the antipiroplasm of *A. herba-alba* against the growth of *B. bovis*, *B. bigemina*, *B. divergens*, *B. caballi*, and *T. equi in vitro*, and *B. microti* in mice.

## Materials and Methods

### Ethical approval

The Animal Care and Use Committee at Obihiro University of Agriculture and Veterinary Medicine approved all of the study’s experimental protocols (Approval No. 27-65). The trials followed the Fundamental Guidelines for the Proper Conduct of Animal Experiments and Related Activities at Academic Research Institutions published by the Ministry of Education (Culture, Sports, Science, and Technology, Japan).

### *In vitro* growth inhibition assay

*A. herba-alba* was dissolved in 50 ml of 99.8% methanol (Wako Pure Chemical Industries, Ltd., Osaka, Japan) and incubated at 30°C for 3 days [18]. The finished product was filtered using Whatman filter paper No. 1 and a rotary evaporator (BUICHI^®^RotavaporR-200/205, Flawil, Switzerland), and a freeze-drying vacuum system (Labconco, Kansas City, MO, USA) [19,20]. The crude extract was then dissolved in dimethyl sulfoxide (DMSO) at 100 mg/ml. *A. herba-alba* methanolic extract toxicity to bovine and equine erythrocytes was assessed using 25 mg/ml as previously published study [21]. 

The chemotherapeutic efficacy of *A. herba-alba* against *B. bovis* (Texas strain) [22], *B. bigemina* (Argentina strain) [23], *B. divergens* (German strain) [24], *B. caballi* [25], and *T. equi* (U.S. Department of Agriculture) [25] was investigated in the current study by a fluorescence assay using a nucleic acid stain SYBR Green I (Lonza, Rockland, ME) [8,22]. The concentrations of *A. herba-alba* utilized ranged from 0.025 to 30 mg/ml. The *in vitro* study used DA, a routinely used antibabesial medication, as a positive control agent with concentrations ranging from 0.1 to 10 µg/ml [22]. Cultures without the drug and cultures with only DMSO (0.3% for *A. herba-alba*) and DDW (0.02% for DA) served as negative experimental controls (Wako Pure Chemical Industries, Ltd., Osaka, Japan). RBCs infected with 1% parasitemia of bovine and equine Babesia/*Theileria* parasites were cultured in 96-well plates for 4 days without daily medium replacement, using 2.5% hematocrit (HCT) for *B. bovis* and *B. bigemina* parasites and 5% HCT for other Babesia and Theileria parasites, as previously established [22,24]. All screened parasites’ *in vitro* regrowth after ceasing A. herba-alba therapy was monitored using a viability assay, as described earlier in our study [24].

The combination therapy of *A. herba-alba* and DA was tested against *in vitro* cultures of bovine Babesia and horse piroplasm parasites having the highest IC50 values, for *B. bovis* and *T. equi*, as previously detailed [6,26]. All in vitro tests were carried out three times.

### In vivo chemotherapeutic effect of A. herba-alba

The *A. herba-alba in vivo* inhibition assay for B. microti (Munich strain) [27] in 25 female BALB/c mice (CLEA Japan, Tokyo, Japan) was performed twice using a fluorescence assay [28]. Five groups of mice (five animals per group) were employed. Simultaneously, with the drug inhibitory effect evaluation, 10 µl of blood was drawn from each mouse’s tail every 4 days to examine hematological parameters using a Celltac MEK-6450 computerized hematology analyzer (Nihon Kohden Corporation, Tokyo, Japan).

### Statistical analysis

A one-way analysis of variance test was used in GraphPad Prism to discover significant differences between the analyzed groups (GraphPad Software, Inc., San Diego, CA). Statistical significance was defined as *p*-value less than 0.05.

## Results

### *Artemisia herba-alba* effectively suppressed the *in vitro* growth of piroplasm

**Figure 1.**
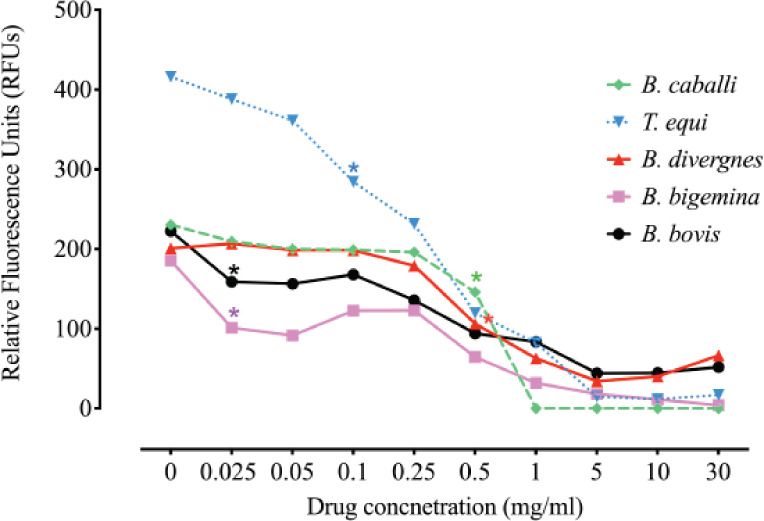
Antipiroplasm efficacy of *Artemisia herba-alba.* Each value represents the mean of three experiments. Asterisks indicate that the treated and control cultures differ significantly (*p *< 0.05).

According to the computed IC_50_s, *A. herba-alba* has the most significant impact on the growth of *T. equi* and *B. bigemina*, followed by *B. bovis* (Table 1). 0.025 mg/ml *A. herba-alba* effectively suppressed the development of *B. bigemina* and *B. bovis in vitro* (*p* < 0.05) (Fig. 1). 0.10 mg/ml *A. herba-alba* was found to be effective in inhibiting the growth of *T. equi* (Fig. 1). Furthermore, 0.50 mg/ml *A. herba-alba* treatments significantly reduced the development of *B. divergens* and *B. caballi* (*p* < 0.05) (Fig. 1).

*Theileria equi* and *B. bigemina in vitro* were suppressed at doses of 0.5 and 1 mg/ml, respectively, in the following viability test (Table 2). The parasite regrowth was suppressed in vitro when B. bovis was given 5 mg/ml *A. herba-alba* (Table 2). With 10 mg/ml *A. herba-alba*, *B. caballi* regrowth began to be reduced (Table 2). The lack of a significant difference (*p* > 0.05) between the DMSO-treated positive control well and the untreated wells shows that the diluent did not affect the efficacy of the *A. herba-alba* methanolic extract. Furthermore, compared to nontreated erythrocytes, pretreatment of erythrocytes with a high dose of *A. herba-alba* methanolic extract at 25 mg/ml did not affect parasite growth pattern or erythrocyte morphology (data not shown).

**Table 1.** IC_50_ values of *Artemisia herba-alba*, diminazene aceturate and other previously used herbal antibabesial drugs evaluated for bovine* Babesia *and equine* Babesia *and *Theileria* parasites

Organism	IC_50_ (µg/ml)^a^			
	*Artemisia herba-alba*	Diminazene aceturate	*Zingiber officinale* rhizome^b^	Turmeric (*Curcuma longa*)^c^
*B. bovis*	412.75±29.05	0.16 ± 0.02	588 ± 23.80	830 ± 78
*B. bigemina*	392.81±31.42	0.08 ± 0.003	14800 ± 1240	ND
*B. divergens*	566.56±37.33	0.046± 0.007	ND	375 ± 55
*T. equi*	303.50±26.50	0.28 ± 0.01	39350 ± 1340	1405 ± 575
*B. caballi*	633.33±34.11	0.012 ± 0.003	356.05 ± 34.71	720 ± 90

a IC_50 _values for *Artemisia herba-alba *and diminazene aceturate were calculated on the fourth day based on the growth inhibitions determined using fluorescence- based assay in three separate experiments. Each drug concentration was made in triplicate in each experiment, and the final obtained IC_50_ represent the mean and standard deviation of three separate experiments. ND, not detected.^ b^ The IC_50_ was reported in previous study (Rizk et al., 2021a).^ c^ The IC_50_ was reported in previous study (Rizk et al., 2021b).

### DA enhanced the in vitro efficacy of *A. herba-alba*

On *B. divergens* and *B. caballi*, different combinations of *A. herba-alba* and DA were tested. On the growth of *B. divergens*, highest concentration of *A. herba-alba* (0.75 IC_50_) demonstrated a synergistic interaction with high doses of DA (0.75 and 0.50 IC_50_) (Table 3). Low doses of *A. herba-alba* had an additive effect with DA in inhibiting the proliferation of bovine Babesia and B. caballi parasites (Table 3). Such findings validated *A. herba-alba’s* potential anti-*B. divergens* effect, especially when given in large doses combined with the regularly used antibabesial medication, DA.

### DA enhanced the in vitro efficacy of *A. herba-alba*

**Figure 2.**
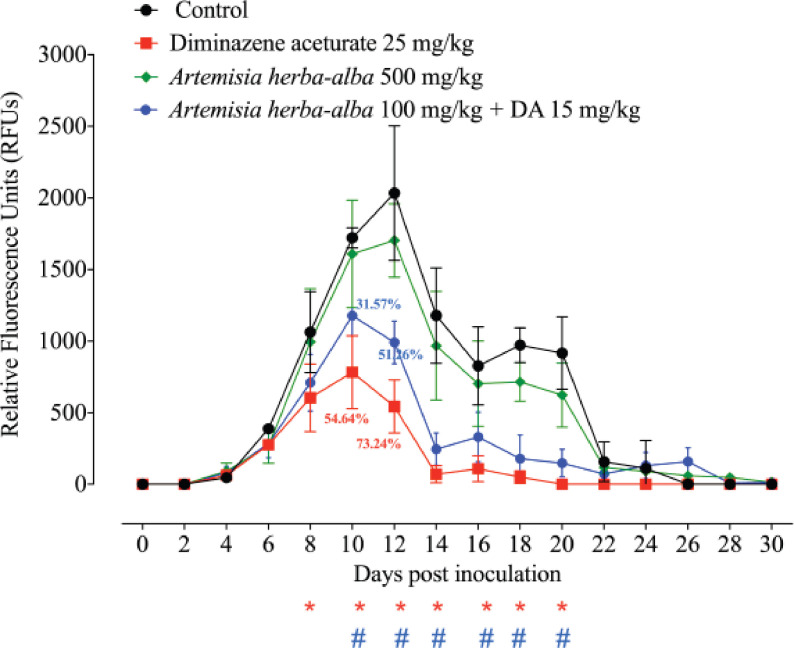
Anti-*B. microti* of *Artemisia herba-alba*. All the animals received 1 × 10^7^ *B. microti* RBCs intraperitoneally. When parasitemia in the infected mice reached about 1%, the treatment began and lasted for 5 days. In the control group, mice were given I/P doses of DMSO in phosphate buffer saline (0.02%). DA and *Artemisia herba-alba* were used in subcutaneous and oral doses, respectively. In the combination therapy, the drugs were given at the same time as the inoculation. The mean and standard deviation of five mice per experimental group are represented by each value. # denotes significant differences (*p *< 0.05) between *Artemisia herba-alba*/DA or DA monotherapy-treated groups and the control group.

*A. herba-alba* was tested in mice for its ability to suppress B. microti *in vivo*. Within the presence of 500 mg kg^−1^ *A. herba-alba* monotherapy, the greatest fluorescence values within the *A. herba-alba*-treated groups reached a mean of 1702 at 12 days p.i. (Fig. 2). At 12 days p.i., 100 mg kg^−1^ *A. herba-alba* with 15 mg kg^−1^ DA demonstrated 991.15 mean fluorescence levels (Fig. 2). The peak fluorescence values in the positive control group, on the other hand, were 2033.65 at 12 days p.i (Fig. 2). Notably, when *A. herba-alba* was given at a low dose of DA, the suppression within the fluorescence values in mice was virtually identical to that shown in mice given 25 mg kg^−1^ DA at peak parasitemia days (Fig. 2). At 10 and 12 days p.i., oral injections of 100 mg kg^−1^ *A. herba-alba* in combination with a subcutaneous dose of 15 mg kg^−1^ DA inhibited parasite growth by 31.57% and 51.26%, respectively, compared to 54.64% and 73.24% inhibitions in the presence of 25 mg kg^−1^ DA (Fig. 2).

**Table 2.** Viability test results of *Artemisia herba-alba* evaluated for *Babesia *and *Theileria* parasite

Drug	Drug concentrations (mg/ml)^a^								
	0.025	0.05	0.1	0.25	0.5	1	5	10	30
*B. bovis*	+	+	+	+	+	+	-	-	-
*B. bigemina*	+	+	+	+	+	-	-	-	-
*B. divergens*	+	+	+	+	+	+	+	-	-
*T. equi*	+	+	+	+	-	-	-	-	-
*B. caballi*	+	+	+	+	+	+	+	-	-

a Each value was calculated using fluorescence assay in three separate experiments. Each concentration of the drug was made in triplicate in each experiment. + = viable; − = dead

**Table 3.** Two druginteractions of *Artemisia herba-alba* in combination with diminazene aceturate on the *in vitro* growth of *Babesia divergens* and *Babesia caballi* parasites

Parasite	C^a^	FIC_D1_	FIC_D2_	ΣFIC	Degree of interaction ^b^
*B. divergens*	0.75 + 0.75	0.21	0.11	0.32	Synergetic
	0.75 + 0.50	0.11	0.21	0.32	Synergetic
	0.75 + 0.25	0.31	0.42	0.73	Additive
	0.50 + 0.75	0.33	0.41	0.74	Additive
	0.50 + 0.50	0.31	0.39	0.7	Additive
	0.50 + 0.25	0.41	0.51	0.92	Additive
	0.25 + 0.75	0.34	0.62	0.96	Additive
	0.25 + 0.50	0.45	0.23	0.68	Additive
	0.25 + 0.25	0.46	0.47	0.93	Additive
*B. caballi*	0.75 + 0.75	0.22	0.41	0.63	Additive
	0.75 + 0.50	0.31	0.55	0.86	Additive
	0.75 + 0.25	0.41	0.33	0.74	Additive
	0.50 + 0.75	0.31	0.45	0.76	Additive
	0.50 + 0.50	0.41	0.41	0.82	Additive
	0.50 + 0.25	0.33	0.41	0.74	Additive
	0.25 + 0.75	0.36	0.38	0.74	Additive
	0.25 + 0.50	0.41	0.33	0.74	Additive
	0.25 + 0.25	0.39	0.44	0.83	Additive

^a^ C refer to the different concentrations of *Artemisia herba-alba* in combination with diminazene aceturate. ^b ^The degree of drug interaction was determined based on the following fractional inhibitory concentration (FIC) index: ≤ 0.5 (synergetic), and > 0.5–1 (additive). FIC_D1 _refers to the fractional inhibitory concentration of *Artemisia herba-alba*. FIC_D2 _refers to the fractional inhibitory concentration of diminazene aceturate. Three independent tests were performed after each combination was loaded in triplicate wells in 96-well plates. FIC_D1= _inhibitory effect of _D1_ in presence of _D2_/ inhibitory effect of _D1_ alone, FIC_D2= _inhibitory effect of _D2_ in presence of _D1_/ inhibitory effect of _D2_ alone, ΣFIC= FIC_D1+_ FIC_D2_

**Figure 3.**
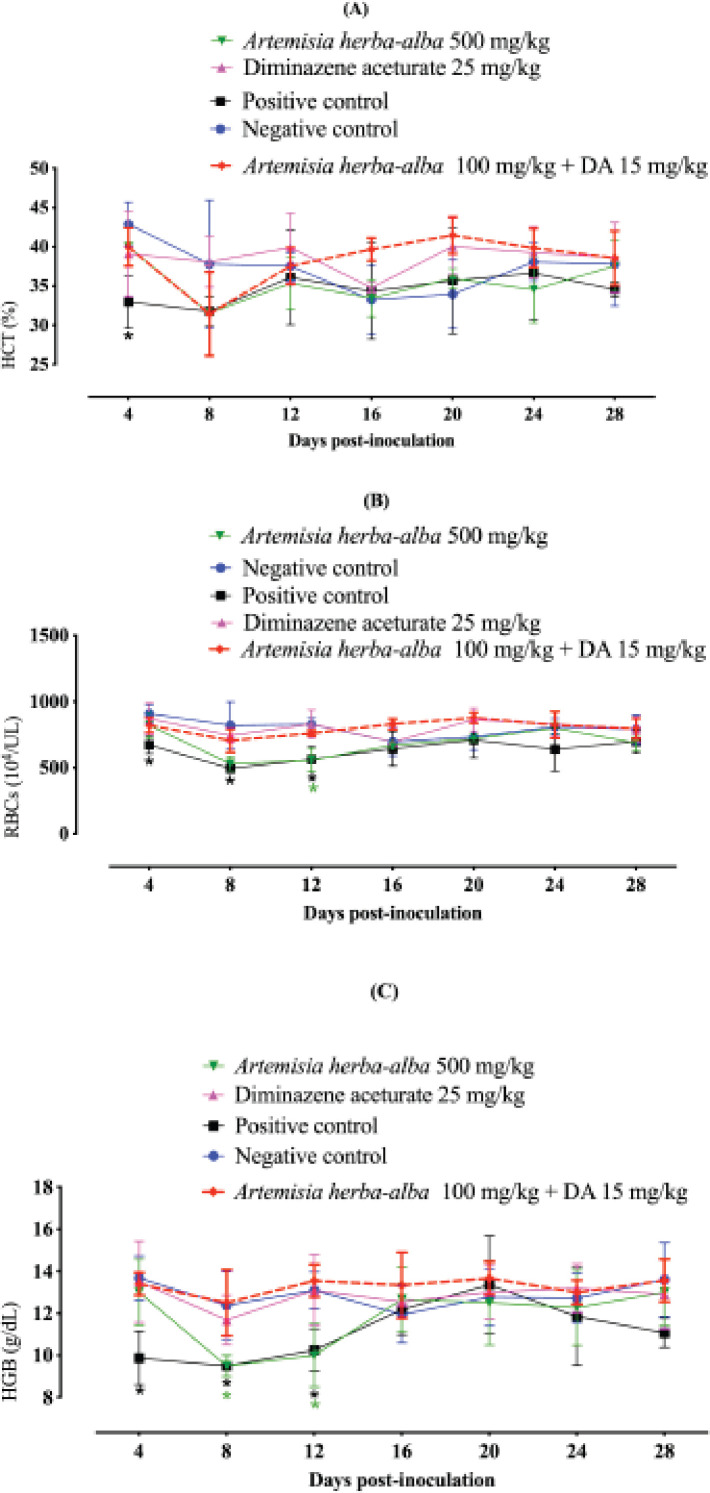
*Artemisia herba-alba* effect on the recovery from anemia associated with *B. microti* infection in mice. (a) HCT. (b) RBCs. (c) Hemoglobin (HGB). Each value represents the mean and standard deviation of five mice per experimental group. Asterisks indicate that the difference between treated or infected animals and uninfected mice is statistically significant (*p* < 0.05).

The use of A. herba-alba in combination with a low DA dose normalized the hematological variables compared to those treated with 25 mg kg^−1^ DA (Fig. 3). These findings indicated *A. herba alba’s* promising antibabesial activity when combined with a low DA dose. Such a regimen may aid in overcoming the toxic effects of high doses of the regularly used antibabesial medication, DA, and the parasite resistance resulting from this agent’s prolonged use.

## Discussion

This study looked at how *A. herba-alba* inhibited the growth of screened piroplasm parasites* in vitro* and *in vivo*. For *B. bovis*, *B. bigemina*, and *T. equi*, *A. herba-alba* had lower IC50 values than *Zingiber officinale* rhizome, a recently identified herbal antibabesial candidate [18]. Similarly, the IC50 for *B. bovis* in *A. herba-alba* was lower than those reported after *in vitro* treatment with turmeric (*Curcuma longa*) [29].

The efficacy of *A. herba-alba* as an agent with antimalarial activity [12,15] may explain the antibabesial efficacy of this herbal therapy because *Plasmodium* and *Babesia *parasites have striking biological similarities. Taken together, the antioxidant effect of *A. herba-alba* [17] may explain the antibabesial efficacy of this medicinal plant owing to the infection by *Babesia* is usually associated with increased levels of free radicals and oxidative stress markers [30], which is harmful to the infected host.

In the current investigation, very high concentrations of *A. herba-alba* exhibited no effect on bovine or horse RBCs. Additionally, *A. herba-alba* has been safely consumed for centuries without adverse effects. Previous studies reported the safe use of *A. herba-alba* in rats at >2 gm/kg [12,31]. Such findings were confirmed via histopathological analysis of animal organs [31]. Interestingly, the 50% lethal dose (LD50) value of *A. herba-alba* in mice was greater than 5,000 mg/kg [32].

The *in vitro* inhibitory activity of *A. herba-alba* and its safety have prompted us to study the inhibitory effect of *A. herba-alba* when taken alone or in combination with DA in mice. In our investigation, the *in vitro* inhibitory effect of *A. herba-alba*, combined with DA, against the growth of B. divergens and B. caballi was strengthened. These results are similar to the *in vitro* inhibitory effects of myrrh oil/DA [21], allicin/DA [33], and thymoquinone/DA combinations [27]. In an *in vivo* study, the inhibition of B. microti growth caused by *A. herba-alba*/DA is nearly similar to 56.35% and 53.25% inhibition rates for 85 mg kg^−1^ PYR combined with 10 mg kg^−1^ DA, respectively [28]. Although the present study evaluated the inhibitory effect of *A. herba-alba* when used as monotherapy or in combination therapy against the growth of B. microti in mice, further studies are required to determine the LD50 of this herbal extract in cattle before its application under field conditions.

## Conclusion

In conclusion, *B. bigemina* and *T. equi* were the most sensitive Babesia species to Artemisia herba-alba’s in vitro inhibitory action, followed by *B. bovis*. *A. herba-alba* was co-administrated with DA, a synergistic interaction against the *in vitro* growth of *B. divergens* was observed. The emitted fluorescence signal in the blood of mice treated with a combination therapy containing lower doses of *A. herba-alba* and DA was significantly reduced. Furthermore, a combination of *A. herba-alba*/DA therapy was used to correct hematological variables and treat hemolytic anemia caused by babesiosis. By overcoming the toxicity and resistance associated with long-term use of the antibabesial drug DA, *A. herba-alba* may be beneficial in treating animal piroplasmosis.

## List of abbreviations

*B. bovis*: *Babesia bovis*; *B. bigemina*: *Babesia bigemina*; *T. equi*: *Theileria equi*; DA: Diminazene aceturate; DMSO: Dimethyl sulfoxide; HCT: Hematocrit; RBCs: Red blood cells; LD_50_: 50% lethal dose.

## Data and materials accessibility

The corresponding author will provide the data sets created and/or analyzed during the current work upon reasonable request.
